# Implementación de la teleconsulta en hospitales de Córdoba Capital durante la pandemia de SARS-CoV-2

**DOI:** 10.31053/1853.0605.v81.n1.42231

**Published:** 2024-03-27

**Authors:** María Virginia Boaglio, Gianmarco Llarena Billia, Ingrid Strusberg

**Affiliations:** 1 Universidad Nacional de Córdoba. Facultad de Ciencias Médicas. Cátedra de Semiología UHMI N°2.; 2 Universidad Nacional de Córdoba. Facultad de Ciencias Médicas. Cátedra de Fisiología Humana; 3 Universidad Nacional de Córdoba. Facultad de Ciencias Médicas. Cátedra de Semiología UHMI N°2. Consejo Académico de Reumatología

**Keywords:** teleconsulta, pandemia, COVID-19, telemedicina, SARS-Cov-2, remote consultation, pandemic, COVID-19, Telemedicine, SARS-Cov-2, teleconsulta, pandemia, COVID-19, telemedicina, SARS-Cov-2

## Abstract

La teleconsulta es una de las prestaciones de la telemedicina, definida como el conjunto de interacciones médico-paciente cuyo fin es el de proporcionar asesoramiento diagnóstico o terapéutico a través de medios electrónicos. El objetivo principal del presente trabajo es estimar la frecuencia de la implementación de la teleconsulta en hospitales de la ciudad de Córdoba durante el periodo 2020-2022. Se realizó un estudio descriptivo, observacional y transversal, brindando una encuesta online (Google Forms) a directivos de los hospitales de Córdoba Capital. Se utilizaron estadísticos descriptivos. Se pudo entrevistar a 30 representantes de los 50 nosocomios que tenía la ciudad, de los cuales 17 eran privados (57%) y 13 públicos (43%). El 82% de los privados ofrecía teleconsulta versus 77% de los públicos. El 92% del total la implementó por la pandemia de SARS-CoV-2 y el otro 8% la ofrecía previamente. Los servicios más demandados fueron Clínica Médica e Infectología. La llamada telefónica fue la herramienta más utilizada. Sobre los nosocomios que no ofrecieron teleconsulta, 67% no planea hacerlo a futuro, y el resto está realizando gestiones para su aplicación en el 2023. La pandemia de SARS-CoV-2 catalizó la implementación de teleconsultas en la mayoría de los hospitales relevados. A pesar de aumentar la accesibilidad a la consulta, aún hay algunos hospitales que no planean incorporar esta modalidad. Contar con estos datos puede constituir una base para mejorar estrategias futuras de atención médica en la ciudad de Córdoba.

## Abstract (en)

A Teleconsultation is one of the assets of telemedicine that allows medical professionals to provide diagnostic or therapeutic advice to patients through electronic means. The objective of this study was to estimate the frequency of teleconsultation implementation in hospitals in the city of Cordoba from 2020 to 2022. A descriptive, observational and cross-sectional study was carried out, providing an online survey (Google Forms) to hospital representatives in Córdoba. Descriptive statistics were used. The study was conducted in 30 out of the 50 hospitals in the city, including 17 private (57%) and 13 public institutions (43%). Teleconsultation was provided by a higher percentage of private healthcare facilities (82%) compared to public ones (77%). The results showed that the SARS-CoV-2 pandemic was the main driver behind the implementation of teleconsultation, with 92% of all hospitals having implemented it due to the pandemic while the remaining 8% had already offered this service prior to the pandemic. The most requested services were Internal Medicine and Infectology, and phone calls were the most commonly used tool. Among the hospitals that did not offer teleconsultation, 67% did not plan to do so in the future, while the remaining 33% were making arrangements for its implementation in 2023. Although the pandemic catalyzed the implementation of teleconsultations in most hospitals, there are still some institutions that do not plan to incorporate this modality. This study provides valuable data for improving future medical care strategies in the city of Cordoba.

## Resumo

A teleconsulta é um dos benefícios da telemedicina, definida como o conjunto de interações médico-paciente cujo objetivo é fornecer aconselhamento diagnóstico ou terapêutico por meio eletrônico. O principal objetivo deste trabalho é estimar a frequência da implementação da teleconsulta em hospitais da cidade de Córdoba durante o período 2020-2022. Foi realizado um estudo descritivo, observacional e transversal, fornecendo uma pesquisa online (Google Forms) para gerentes de hospitais em Córdoba Capital. Estatísticas descritivas foram usadas. Foi possível entrevistar 30 representantes dos 50 hospitais que a cidade possuía, sendo 17 privados (57%) e 13 públicos (43%). 82% dos privados ofereciam teleconsulta contra 77% dos públicos. Do total, 92% o implementaram devido à pandemia de SARS-CoV-2 e os outros 8% o ofereceram anteriormente. Os serviços mais procurados foram Clínica Médica e Infectologia. O telefonema foi a ferramenta mais utilizada. Quanto aos hospitais que não ofereceram teleconsulta, 67% não planejam fazê-lo no futuro, e os demais estão se preparando para aplicá-la em 2023. A pandemia de SARS-CoV-2 catalisou a implementação de teleconsultas na maioria dos pesquisados hospitais. Apesar de aumentar a acessibilidade à consulta, ainda existem alguns hospitais que não pretendem incorporar esta modalidade. Ter esses dados pode constituir uma base para melhorar as futuras estratégias de atendimento médico na cidade de Córdoba.

**CONCEPTOS CLAVE**

**Qué se sabe sobre el tema.**

La utilización de la teleconsulta se incrementó durante la crisis sanitaria del COVID-19 como una alternativa globalmente utilizada para disminuir riesgos de exposición de los pacientes durante la atención médica. Sin embargo, no se cuentan con datos estadísticos respecto a su implementación en la Ciudad de Córdoba, Argentina.

**Qué aporta este trabajo.**

En este estudio descriptivo, observacional y transversal realizado en los nosocomios de la Ciudad de Córdoba, se presentan datos y conclusiones sobre la implementación de la teleconsulta en la pandemia de COVID-19, durante el periodo entre marzo 2020 y abril 2022.

**Divulgación**

La utilización de la teleconsulta se incrementó durante la crisis sanitaria del COVID-19 como una alternativa globalmente utilizada para disminuir riesgos de exposición de los pacientes durante la atención médica. Sin embargo, no se cuentan con datos estadísticos respecto a su implementación en la Ciudad de Córdoba, Argentina. En este estudio descriptivo, observacional y transversal realizado en los nosocomios de la Ciudad de Córdoba, se presentan datos y conclusiones sobre la implementación de la teleconsulta en la pandemia de COVID-19, durante el periodo entre marzo 2020 y abril 2022.

## Introducción

La Organización Mundial de la Salud (OMS) define a la telemedicina como "la prestación de servicios de salud (en los que la distancia es un factor determinante) por parte de profesionales de la salud a través de la utilización de tecnologías de la información y la comunicación para el intercambio de información válida para el diagnóstico, el tratamiento, la prevención de enfermedades, la investigación, la evaluación y para la formación continua de profesionales de la salud, todo ello con el objetivo final de mejorar la salud de la población y de las comunidades"**.** La Organización Panamericana de la Salud (OPS), se propicia activamente la telemedicina, hecho de gran relevancia para la salud pública en países que la conforman, entre ellos Argentina, no sólo porque la telemedicina presenta un crecimiento meteórico, sino también por su significativo potencial para lograr mejorar la eficiencia y equidad en el ejercicio de un derecho básico y fundamental como es el acceso de la población al Sistema de Salud ^1^

La telemedicina se utiliza en prestaciones como la educación de grado y capacitación a distancia y continua de profesionales de la salud hasta la aplicación de sistemas informáticos en hospitales. Una de dichas prestaciones, y sobre la cual se trata el presente trabajo, es la teleconsulta o teleasistencia. Ésta es definida por la OPS como el conjunto de interacciones que ocurren entre un médico y un paciente con el fin de proporcionar asesoramiento diagnóstico o terapéutico a través de medios electrónicos ^2^ . La teleconsulta incluye el telediagnóstico, la teleconferencia y el almacenamiento digital de datos o fichas electrónicas.

En 2020, la teleconsulta permitió que los pacientes con síntomas compatibles con COVID-19 fueran evaluados en triaje de manera personalizada sin asistir al centro de salud, siendo indicación el aislamiento en el hogar si estaban fuera de riesgo, y la derivación al servicio de emergencias en caso contrario, protegiendo así al resto de la exposición al virus. También permitió que médicos y pacientes se comunicaran 24 horas del día, los 7 días de la semana, mediante teléfonos inteligentes o computadoras con cámara web^3^.

En Argentina, el Ministerio de Salud aprobó en 2019, la 1ª Recomendación para el Uso de la Telemedicina (Resolución Nº 21/2019). Este documento, que se confeccionó dentro del marco de la Estrategia Nacional de Salud Digital 2018-2024, sostiene como uno de sus objetivos principales, que se implementen redes de telesalud que permitan la atención a distancia del paciente y las consultas de segunda opinión, mejorando la accesibilidad, evitando traslados y compensando las diferencias regionales de especialidades y recursos ^4^.

A agosto 2023, no existe un marco legal en Argentina que regule la telemedicina, aunque se han propuesto varios proyectos de ley^5^. La ley 25.326, conocida como Ley de Protección de Datos Personales o Hábeas Data, protege los datos de identidad - de salud entre otros- para evitar que sean usados sin consentimiento. Por lo tanto, sería imprescindible que las teleconsultas garantizaran su cumplimiento^6^. Con el advenimiento de la pandemia de SARS-CoV-2, el Decreto 297/2020 del Poder Ejecutivo Nacional estableció el Aislamiento Social Preventivo y Obligatorio (ASPO). Las urgencias surgidas en la pandemia motivaron el dictado de la Resolución de la Superintendencia de Servicios de Salud de la Nación Nº 282/2020^7^ y la implementación del programa Tele-Covid 3, por medio de los cuales se comenzó a fomentar el uso de plataformas de teleasistencia y/o teleconsulta.

En agosto de 2020 se aprobó la Ley Nº 27553 de Receta Electrónica y Teleasistencia en Salud, que habilitó la teleasistencia para el ejercicio de la medicina. Dos meses después, el Senado dio media sanción al proyecto de Ley de Telemedicina. Dicho proyecto se basa en los principios éticos de universalidad del acceso a los servicios de salud, accesibilidad a una medicina oportuna y de calidad, seguridad de los datos sensibles, eficiencia en el uso de los recursos disponibles, descentralización del sistema sanitario, confidencialidad en la relación médico-paciente y equidad^8^. En la Provincia de Córdoba, el gobierno instauró en 2018 el Programa "Sí Salud" en los hospitales públicos provinciales con el objetivo de modernizar los procesos de atención y gestión hospitalaria en toda la red provincial de salud. Una de las nuevas prácticas ofrecidas por el programa es la telemedicina^9^.

El objetivo principal del presente trabajo es caracterizar la implementación de la teleconsulta debido a la pandemia por SARS-CoV-2 en 30 hospitales de la ciudad de Córdoba. Entre los objetivos secundarios se busca establecer qué servicios hospitalarios pusieron en práctica la teleconsulta, determinar si las teleconsultas aplicadas por los diferentes hospitales se empezaron a realizar desde el momento en que se decretó la pandemia de SARS-Cov-2 en Argentina o previo a ésta, analizar diferencias entre el ámbito público y privado con respecto a la frecuencia de implementación de las teleconsultas, precisar si los servicios médicos que la aplican realizan estadísticas sobre las mismas, establecer la satisfacción con respecto a su uso y aplicación, estimar cuántos hospitales de los que aún no la han implementado tienen previsto emplearla en el futuro.

## Materiales y Métodos

Se realizó un estudio descriptivo, observacional y transversal, mediante encuesta no validada, online, a través de un formulario Google Forms, enviado por email o WhatsApp a directivos o representantes de 50 hospitales de Córdoba entre agosto 2021 y abril 2022. Consistió de 13 preguntas estructuradas, de opción múltiple única y otras semiestructuradas formuladas por los autores de la investigación en base a los objetivos planteados. La tasa de respuesta fue del 60% (n=30). Cada institución se consideró la unidad de análisis. Previo al envío de la encuesta, el consentimiento informado fue obtenido de manera verbal. Se aclaró que todos los datos recopilados y publicados se presentarían de manera anónima y solo se utilizarían con fines relacionados con esta investigación. El llenado del formulario constituyó el consentimiento para la participación en el estudio. El proyecto había sido aprobado por el Comité Institucional de Ética del Hospital Nacional de Clínicas y la Secretaría de Ciencia y Tecnología- ambos de la Facultad de Ciencias Médicas de la Universidad Nacional de Córdoba. Todas las variables se expresaron en valores absolutos y porcentajes. Se utilizó Microsoft Excel para realizar el mismo.

## Resultados

### Implementación de teleconsultas:

Entre agosto de 2021 y abril de 2022 se entrevistó a 30 profesionales que contestaron en carácter de representación de distintos hospitales de la ciudad de Córdoba. Un 56,67% (n=17) fueron hombres, 17 hospitales pertenecieron al ámbito privado (56,67%), 10 al servicio público provincial (33,33%), 2 al servicio público nacional (6,67%) y 1 al servicio público municipal (3,33%). De los 20 centros hospitalarios que no respondieron, 65% (n=13) pertenecen al ámbito privado y 35% (n=7) al ámbito público. El 80% (n=24) de los 30 centros hospitalarios que respondieron la encuesta, contaban con servicios de teleconsulta; 14 del ámbito privado y 10 del ámbito público (Fig. 1). Se preguntó si en relación con el momento del comienzo de la implementación de las teleconsultas, las mismas se aplicaban previo a la aparición de la pandemia por SARS-Cov-2 o a causa de la situación desencadenada por la pandemia. De ellos 91,66% (n=22) implementaron dicha asistencia a raíz de la situación desencadenada por la pandemia de SARS-CoV-2 y el otro 8,3% (n=2) la ofrecía antes de la situación sanitaria de emergencia. El 20% (n=6) restante no brindaba dicha prestación.

**Figura Nº 1:**
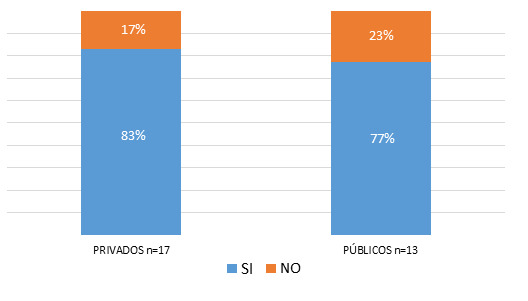
Proporción en la implementación de la teleconsulta según ámbito privado y público.

Si bien no se interrogó el detalle de todos los servicios que cada hospital ofrecía, sí se encuestó en relación con los servicios que implementaron la teleconsulta (Tabla 1). De éstos ultimos se encuestó acerca de la existencia o no de análisis de datos. Para llevar un seguimiento dentro de cada servicio, un 58,33% (n=14) de los 24 centros que ofrecían teleconsultas llevaba controles estadísticos sobre las mismas.

**Tabla Nº1:** Cantidad de hospitales que implementaron la teleconsulta según cada servicio.

**Servicio**	**Cantidad de hospitales**
Clínica médica	12
Infectología	11
Cardiología	9
Diabetología	8
Alergia e inmunología; Clínica dermatológica; Neumología; Pediatría.	7
Cirugía; Gastroenterología; Nefrología; Neurología; Oncología; Salud social; Urología.	6
Medicina general y familiar; Otorrinolaringología; Reumatología	5
Hemoterapia e inmunohematología; Medicina del deporte; Ortopedia y traumatología; Terapia intensiva	4
Diagnóstico por imágenes; Endocrinología; Ginecología; Epidemiología	2
Medicina del trabajo; Medicina legal; Patología	1

Los centros médicos encuestados utilizaron distintas herramientas para realizar las teleconsultas, siendo la más utilizada, la llamada telefónica por un 41,50% de los participantes (Fig. 2).

**Figura Nº 2.**
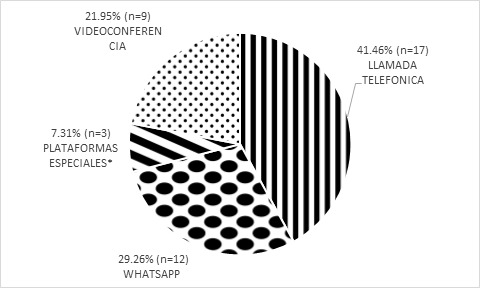
Herramientas utilizadas para las teleconsultas. *Plataformas especiales: programas propios de las instituciones desarrollados para confección y archivo de historias clínicas.

### Satisfacción sobre la aplicación de la teleconsulta:

Se encuestó sobre algunos indicadores de eficacia, y el 75% (n=18) refirió como el más representativo al que se expresó como "Aumento en la cantidad de pacientes atendidos en comparación a los revisados previamente a la utilización de la teleconsulta" y el 25% (n=6) eligió "Aumento en el número de personas con discapacidades o limitaciones físicas, económicas, etc. que a partir del programa han tenido acceso a una atención especializada".

Se indagó sobre la utilidad que ofreció la telemedicina en cada centro médico. El 87,50% (n= 21) estimó que este tipo de asistencia le era de utilidad; 4,17% (n= 1) creyó que fue útil, pero solamente por la situación sanitaria de emergencia de la pandemia de SARS-CoV-2; 4,17 % (n=1) opinó que su rentabilidad se aplicaba sólo a algunas especialidades y 4,17% (n=1) comentó que utilizó el servicio con fines de vigilancia epidemiológica.

### Centros hospitalarios que no ofrecen teleconsultas:

Sobre el total de los nosocomios encuestados que no ofrecían este tipo de prestación a distancia (n=6), 66.67% (n=4) no planeaban su implementación a futuro, mientras que el resto estaban realizando gestiones para su aplicación en el próximo año (n=2). En estas instituciones no se ofrecía la teleconsulta debido a que un 50% (n=3) estaba en desacuerdo con la utilidad de la misma, un 16,66% (n=1) consideraba que no estaba dentro de las prioridades y existían falta de recursos humanos, y 16,66% (n=1) era un hospital escuela que necesitaba y exigía la presencialidad para cumplir de manera eficaz su función de enseñanza.

## Discusión

La pandemia originada por el coronavirus SARS-CoV-2 marcó un antes y un después en la historia de la telemedicina. La necesidad de establecer un canal de comunicación entre pacientes y médicos aceleró la disponibilidad e impulsó la adopción de la teleconsulta por profesionales de todo el mundo a partir del 2020. Surgió como complemento de la consulta presencial proporcionando rapidez en la evacuación de inquietudes, detección precoz de la necesidad de evaluación por el sistema de emergencias, seguimiento del tratamiento, entre otras.

Este trabajo relevó 30 hospitales de la ciudad de Córdoba. De los 24 que ofrecen teleconsultas, 22 las implementaron como respuesta a la aumentada necesidad de atención clínica durante el 2020. Las especialidades que más adhirieron a esta práctica fueron Clínica Médica, Infectología y Cardiología. En contraposición, Anatomía Patológica, Medicina Legal y Medicina del Trabajo fueron las especialidades en las que se vio la menor implementación de la modalidad a distancia. Esto puede haberse debido a que, por la situación de ASPO disminuyó la demanda^10^.

Se invitó a responder la encuesta a lo largo de 6 meses a los 50 hospitales existentes en la ciudad de Córdoba, no obteniendo respuesta de 20 de ellos. Esto podría deberse a que, en un primer momento, debido al ASPO, no se pudo acceder a otro método de comunicación que no fuera el virtual sumado al poco tiempo disponible de los profesionales de la salud. Luego, la llegada de nuevas olas que estresaron el sistema, podrían haber dificultado disponer de tiempo para responder. La muestra representó el 60% del total con una distribución pareja entre el ámbito público y privado.

Como fortaleza de este trabajo se debe tener en cuenta que aún no hay datos publicados sobre la frecuencia de la implementación de teleconsultas en Córdoba Capital desde el inicio de la emergencia sanitaria. La telemedicina es uno de los "legados" de la pandemia y pese al decreto del fin de la misma en mayo 2023 por parte de la OMS, aún continúa utilizándose globalmente^11^. Dos de las instituciones en las cuales aún no se está aplicando han confirmado que sí lo harán.

En el presente trabajo se optó por encuestar al director/a de la institución o a quien éste designara como representante. Una mejora en la metodología utilizada, para evitar el sesgo de representatividad, hubiera sido ampliar la muestra a cada jefe de servicio. Nuestros resultados mostraron que casi el 60% de los centros que ofrecían teleconsultas llevaban estadísticas de las mismas. Sin embargo, es posible que ese porcentaje fuera mayor y que, en realidad, el representante a la hora de responder, no haya contado con toda la información la totalidad de los servicios que estaban utilizando telemedicina.

En un estudio realizado a 76 trabajadores del área de salud mental en 5 jurisdicciones del país, se vio que al igual que en otros servicios, la conducta presencial se reservó para situaciones de urgencia o emergencia. En el resto de las consultas existió la alternativa para seguimiento o envío de recetas mediante plataformas digitales o llamada telefónica, destacando positivamente esta última para el contacto con familiares de los pacientes psiquiátricos^12^. La ausencia de datos en nuestro estudio obedece a que no hubo respuesta al intentar contactarnos con los directivos de instituciones de salud mental.

Si bien hay poca diferencia entre la cantidad de hospitales públicos y privados que fueron encuestados, en nuestra investigación se observó que los centros hospitalarios públicos presentaron una menor implementación de la teleconsulta que los centros privados. Como posibles motivos se podría considerar una menor disponibilidad de recursos tecnológicos y económicos en los centros de salud públicos. Sin embargo, las decisiones gerenciales en ambos medios habrían tenido un rol importante para permitir la inversión de recursos que mejoren la disponibilidad de la telemedicina. Resultó llamativo que un hospital escuela relevado contestó que por su condición de tal no implementó ni iba a implementar esta modalidad. Es posible que esto constituyera una opinión personal ya que fue alguien delegado por el director de esa institución. Es en este tipo de hospitales, en donde la educación debería incorporar cambios innovadores que acompañan a los hitos en la historia de la salud humana.

En el año 2021 en un hospital nacional de Lima, Perú, se desarrolló una tesis en la que se concluyó que implementar un sistema de teleconsultas para pacientes crónicos ambulatorios era sumamente necesario, con el fin de no exponerlos durante la emergencia sanitaria^13^. Un estudio realizado en Argentina en los comienzos de la pandemia, concluyó que existió un daño en el cuidado de enfermedades no infecciosas tales como las cardiovasculares, cerebrovasculares y oncológicas. Se evidenció un pronunciado descenso en consultas de emergencia y hospitalizaciones desde marzo 2020^14^. Ese daño colateral descripto previamente, parecería contrastar con los resultados obtenidos en nuestro trabajo ya que 75% de los encuestados respondió que existió un aumento en la cantidad de pacientes atendidos en comparación a los revisados previamente a la utilización de la teleconsulta.

El 90% de nuestros entrevistados consideraron que fue útil la implementación de la teleconsulta. Ya en 2018 una investigación en la Universidad privada Norbert Wiener de Lima, describía que la telemedicina brinda el beneficio de ofrecer atención especializada a pacientes ubicados en áreas alejadas o de difícil acceso, y que al mismo tiempo contribuye a la organización y descongestionamiento de los hospitales^15^. Por eso, también es evidente que en pacientes con enfermedades crónicas que se encuentran con las dificultades mencionadas para acudir a una consulta presencial, siempre y cuando el motivo de consulta no requiera realizar un examen físico; sustituir ésta por la teleconsulta, y la receta física por una receta electrónica parece ser una solución acertada.

Casi la mitad de las teleconsultas se llevaron a cabo por medios tecnológicos diferentes a la llamada telefónica. Es importante que los adultos mayores logren una mayor integración digital debido, no solo a que son una de las poblaciones más vulnerables, sino que podría evitarles el salir de sus hogares en casos de imposibilidad o dificultad física y/o económica para trasladarse. Para promover la accesibilidad universal a los servicios de salud se requiere contrarrestar la brecha digital contemplando tres factores: el acceso a los recursos tecnológicos, la conectividad necesaria para lograr la comunicación y las habilidades para interactuar con la tecnología ^16^ **.**Aun, a pesar de todo lo expresado, no existen guías creadas por consenso de expertos o por sociedades para atender vía digital y definir los pasos y las tecnologías aceptables para una teleconsulta.

Las instituciones de salud de Córdoba durante la pandemia mostraron capacidad y voluntad para implementar herramientas de telemedicina y facilitar la consulta médica. En la actualidad una de las limitantes para continuar con ello es la prescripción de medicamentos. La Ley de Recetas electrónicas 27.553 fue sancionada en Argentina en agosto del 2020^8^. Desde el mes de abril de 2020, en Argentina se aprobó por decreto del Ministerio de Salud la implementación de la "foto receta" para el expendio de fármacos. Este tipo de prescripción, fue uno de los avances en la aplicación de la teleconsulta en el país, ya que, para ese momento, muy pocos profesionales tenían su firma digital registrada, o la posibilidad de realizar receta electrónica. La principal desventaja de este sistema, que estuvo vigente hasta diciembre de 2022 con extensión hasta el 28 de febrero de 2023 solo para pacientes con enfermedades crónicas, fue la sobreprescripción y la automedicación. Era factible falsificar recetas con medios digitales de edición e imposible rastrear si la receta fue utilizada varias veces en distintas farmacias^17^. Una posible solución al problema planteado, sería incentivar a los profesionales a registrar su firma digital, y a los financiadores de salud -sean públicos o privados- a implementar la receta electrónica para poder continuar con las teleconsultas dentro de un marco legal. Es posible que aún no haya sido implementado por los costes que implica reformar los sistemas informáticos tanto en los financiadores de salud como en las instituciones prestadoras.

## Conclusión

La pandemia de SARS-CoV-2 catalizó la implementación de teleconsultas en la mayoría de los hospitales relevados de la Ciudad de Córdoba. Los servicios más demandados fueron Clínica Médica e Infectología. La llamada telefónica fue la herramienta más utilizada. A pesar de aumentar la accesibilidad a la consulta, aún hay algunos hospitales que no planean incorporar esta modalidad. Contar con estos datos puede constituir una base para mejorar estrategias futuras de atención médica en la ciudad de Córdoba.
